# The Doctor as Expert and Pioneer
1Read before the Bath and Bristol Branch of the British Medical Association, April 25th, 1906.


**Published:** 1906-09

**Authors:** Charles J. Whitby


### THE DOCTOR AS EXPERT AND PIONEER.[1]

BY

CHARLES J. WHITBY, M.D.

THERE can, I think, be no doubt that for the public at large the medical profession functions as the type or symbol of the science of the day. And the reason of this is not far to seek. The electrical or mechanical engineer, the naval architect, the analytical chemist or the agricultural expert, may have equal or even greater claims to pose as the representatives of applied science, but, inasmuch as their work does not bring them into personal touch with society, their influence on public opinion is comparatively small. But the doctor in his daily round enters the house of prince and of pauper; he knows and is known by everybody, and his lightest word is the word of an initiate rendered authoritative by his accredited access to the latest spoils of contemporary research. The deference we are accustomed to expect, and upon the whole to receive, is not merely a personal deference, it is also the allegiance of uninstructed to expert opinion, to an impersonal authority whose mouthpiece we are or profess to be. If our confident predictions are falsified, our advice proved untrustworthy, our treatment disastrous, it is not we only whose reputations are impaired. By our failures the glory of Science herself, whose methods we claim to exemplify, is brought into question, and sometimes into ridicule or denial. By our successes, not few or of small account, I hope, the rightful supremacy of those methods is vividly enforced. So it is that in a given community the good or ill repute of our profession is a fair index to the condition and prospects of Science in general.

The economic support of the public is an essential condition of scientific progress, and will no doubt be favourably or adversely affected by the good or bad impression which we, as

[1] Read before the Bath and Bristol Branch of the British Medical Association, April 25th, 1906.

the accepted representatives of scientific method, produce on the minds of our patients. An interesting point in this connection is the influence of democratic ideas and institutions on the position and authority of the expert. The typical democrat is, as a rule, somewhat jealous of the claims of the expert, he regards them with suspicion, defers to them grudgingly if at all, and without perhaps in the least understanding the matter, is apt to proclaim disbelief in their validity with a confidence worthy of a better cause. A certain amount of moral and intellectual insubordination, an all-round scepticism, is a logical correlative of democratic tendencies, and the doctor, in common with other authorities, has in these days to be prepared to justify his proceedings at every turn. He cannot vaccinate a baby without first submitting to cross-examination on the statistics of variolous immunity, or administer a dose of calomel without allaying the terrors of the anti-mercurial zealot. Your ultra-radical will insist that his primiparous wife must be delivered by "natural" means only; he "does not believe" in instrumental interference or the use of anæsthetics. I make these observations, the truth of which your experience will doubtless compel you to confirm, with no intention of belittling popular government, but as a simple statement of fact. Science does not fear criticism, but welcomes it, and thrives upon it as we know. A critical atmosphere hardens and fortifies the spirit of research, as cold pure air braces the human organism. But captious quibbling and prejudiced question-begging are of course another matter, and of them too we have enough and to spare. In some ways an autocracy, or such a *régime* of strong centralised government as obtains now in Germany, is doubtless more favourable to the just authority of the expert than our own more liberal constitution. On the other hand, we must always remember that science is made for man, not man for science. In other words, we must not disdain to justify our assertions when challenged by ignorance or mistrust, since what we desire is not sullen acquiescence but willing and intelligent co-operation. For we too are fallible, and have, in fact, if we know the history of our calling, to admit conviction of not a few gross errors very confidently and unanimously proclaimed as indubitable truths in their day. The expert, *quâ* expert, is a man of his own generation, sharing not only its knowledge and skill but also its prejudices and limitations. What he sees he sees very clearly, what he does not see he is too apt to ignore, or somewhat rashly and prematurely to deny. In so doing he runs the risk of being falsified by the event, for the vague possibilities of to-day are often the platitudinous verities of to-morrow.

Consider, for example, the astonishing revolution which has overtaken medical opinion as to the rôle of alcohol in health and disease. Fifty years ago we were practically unanimous in asserting the value, nay, even the necessity of stimulants for those who enjoyed and would continue to enjoy good health, while in almost every disease which we were called upon to treat they were freely and fearlessly prescribed. The question in those days was not of alcohol or no alcohol, but merely in what form and how much. Only the other day I saw quoted in a daily newspaper the no doubt self-forgotten dictum of a great and justly revered physiologist, still amongst us, to the effect that no man could enjoy real health and strength without the regular use of alcoholic beverages. In saying this the physiologist in question did not really speak as an expert, but uttered a dogmatic, and, as it happens, erroneous opinion. But the public would of course conclude at the time that so definite an assertion, coming from an acknowledged authority upon the subject of health and its conditions, was based upon substantial and verifiable grounds.

The expert owes it not only to himself but to science to distinguish clearly in his own mind, and above all in his utterance, between those conclusions which are firmly based upon irrefutable evidence and those which are mere opinions awaiting the verdict of time. Strictly speaking, there is in the scientific sphere no recognition of the claims of authority, every result stands or falls according to the objective quality of the evidence available in its behalf. A very good corrective of the narrowing tendency of the specialising expert's work is the due cultivation of what is called the historic spirit. If we to-day are convinced that alcohol is very far from being a necessity of life, we are only returning to the normal common-sense view of enlightened people in most times and places. Two thousand years ago Julius Cæsar, finding in his conquest of Gaul no tougher or more formidable opponents than the tribe of the Nervii, noted with characteristic appreciation the fact that they were water drinkers, who, on account of its injurious effects on their sinews and courage, forbade wine to be brought among them.

Most of us can remember in our student days having puzzled our heads over elaborate and strangely-contradictory statements as to the number of grammes of proteid, amyloid, and fatty material *per diem* necessary to maintain a normal human adult in health and activity. Personally I confess to having regarded these tables with a good deal of suspicion, partly because of their obvious mutual inconsistency, and partly on account of the crudity of the methods of investigation upon which they were based. One well-known table was drawn up by a celebrated German physiologist, who found that it represented the quantity and proportions of food upon which *he* felt well. It was copied from text-book to text-book, and became a sort of classic—an authoritative statement of the needs of the human organism. That it was entirely erroneous I need scarcely say, inasmuch as Prof. Chittenden, by a series of careful experiments, has recently demonstrated that many people can fully maintain the nutrition and functional activity of the body on a quantity of food which would, before these experiments had proved the contrary, have been considered miserably inadequate. An instructive instance of the danger of passing off as matter of expert knowledge what is in truth mere matter of unverified opinion.

It behoves us in these days to be the more careful as to the scientific validity of our official statements because of the freedom with which irresponsible persons, and in particular that modern idol the literary man, delivers himself of cocksure conclusions upon things as to which he has no real knowledge at all. Take the flagrant case of that notorious publicist, Mr. George Bernard Shaw. Mr. Shaw calls himself a Socialist, and the creed of the Socialist presumably includes a due

16

respect for conscientious work of all necessary kinds. That the work of the general practitioner is necessary perhaps even Mr. Shaw would not venture to deny. That it is laborious and exacting we all know pretty well. In what way is a man who merely writes plays qualified thereby to act as a censor of men who attend to the sick and suffering? This oracle has the impertinence to sneer at the "inculcated erroneousness of the general practitioner." The world could more easily dispense with Mr. Shaw's plays than with the work of nine out of any ten of those men for whose intelligence he affects an easy scorn. Then after giving away the case against vaccination by admitting that the possibility of artificial immunisation is still an *open* one (as if Jenner, Pasteur and Behring had never lived), Mr. Shaw proceeds in desperation to attack the technique of the vaccinator (a mere "general practitioner"), and to assert in his usual *ex cathedrâ* style of infallibility that vaccination is "attempted murder," and that its results are precisely the same as those of rubbing the contents of the dustbin into an open wound. These inaccurate statements of course found ready acceptance in the columns of the *Daily News*, and were no doubt duly relished by its readers. Vaccination *is* unpleasant, and how eagerly at all times are acclaimed the most puerile arguments for the neglect of unpleasant obligations. Mr. Shaw, like all egoists, fails by reason of his mean estimate of other folks' ability. Even for the immortal *doyen* of his own calling he has practically no commendation to spare, so it is hardly surprising that our profession is included within the ban of his general censoriousness.

"He who can does, he who cannot teaches." So by the mouth of one of his *dramatis personæ* epigrammatises the author of *Man and Superman*. And it is for once a true though a self-condemnatory maxim. The intellectual influence of our profession is, I am proud to think, securely based on the fact that its tasks keep us firmly and steadfastly in touch with commonplace realities. We are perforce not mere scholars but artisans also; brain and hand are developed together, as in the evolution of our species we are taught that Nature ordained from the first. This it is which keeps or should keep us progressive, which in the long run will render us more than a match for the men of words, who think to find facts as pliable as they find the fantastic incidents of their plays and novels, and with men of sound judgment will render our verdict more acceptable than that of our flimsy opponents.

Yet there is need of vigilance, for the name of the enemy is Legion, and his wiles are many and specious. Take the question of quackery, and of the impudent lies complacently published on its behalf by respectable journals. I read in the paper that Mr. Herbert Gladstone, a member of our enlightened and progressive Government, in answer to a question by Mr. Myer, admitted that the sale of quack medicines was an evil, but considered that it was to be met rather by the spread of education than by Government prosecutions for fraud. The clear implication is that only the uneducated are liable to beguilement by the syren song of the patent pill factor and the "strong man" turned charlatan or self-styled healer. Is it just or even decent that men who have submitted to a long, arduous, and costly training, who from motives of honour and public spirit refrain from self-adulation or display, who share all their hard-won knowledge, not even concealing the steps by which it is being or is hoped to be attained, shall be liable to the irresponsible competition of presumptuous and ignorant braggarts? The gods help those who help themselves. The public takes good care that the qualified doctor is a genuine expert, but it cares little to protect him from the parasites who trade upon average credulity and slander us daily in lying advertisements. If we wish these things altered we must see to it ourselves, there is no other way.

The plague of amateur doctors will never be stayed by mere education. On the contrary, it is perhaps the educated section of the community who both actively and passively are the worst sinners in this respect. Your modern quack is no mere ignoramus; he has often received a University education, and he poses as a reformer of our therapeutic and dietetic iniquities. He figures largely in the Press, both as the munificent source of costly advertisements and as a paid contributor of pseudo-scientific disquisitions. His portrait confronts us in the pages of English and American magazines, modestly chaperoned by those of the greatest scientific discoverers of the present and past ages. His massive torso, represented in an attitude of profound meditation upon the merits of the *dernier cri* in beef extracts or tonic elixirs, adorns the window of the ready-money druggist.

It must of course be admitted that legally-qualified men have no absolute monopoly of discovery even in their own field. The inventor of the laryngoscope was not a medical man but a great singer. Pasteur, the true founder of bacteriology and serum-therapy, which is as much as to say the revolutionist of medical science, was a layman. Our present attitude with regard to alcohol was in great measure foreshadowed by a popular movement, which proclaimed, perhaps prematurely and without scientific justification, yet as it proved correctly, the fallacy of our official view in those days. What we now call suggestion was, under the name of mesmerism, for long the happy hunting-ground of sciolists and quacks, while therapeutic electricity is even yet fighting for that position within the professional fold from which it has too long been unwarrantably excluded. It is in view of such facts of recent history—awkward facts they might be called—that I would modestly urge that the doctor *quâ* expert needs to be supplemented and completed by the doctor *quâ* pioneer. Let me suggest as a theme for exercise in the art of social prognosis the consideration of the ultimate effect of our knowledge of Japanese energy and efficiency on the one hand and of Japanese abstemiousness in respect of animal foods on the other upon our time-honoured national faith in the supreme fighting and working qualities of the largest consumers of Old England's roast beef.

One matter in which our profession may fairly claim to have played the part of social pioneers is the introduction of motor vehicles. No class of the community has been quicker to realise and avail itself of the advantages of petrol horse-power over mere horse-flesh. I look upon the advent of the motor as an innovation almost equal in importance to that of the steam locomotive; not so much perhaps for its direct and immediate consequences, as for those remoter but not less inevitable results which I foresee. The dust problem, for example, has at last been definitively and even substantially raised, and I think it may fairly be assumed that its final solution is merely a matter of time. The abolition of dust would, in my opinion, be a hygienic reform in comparison with which the elimination of the malaria-bearing anopheles would appear almost insignificant. The desiccated filth which blows about our streets and enters our houses by every nook and cranny, swarming no doubt with virulent microbes of every description, is, I am convinced, responsible for a much larger proportion of minor and major ailments than is at all commonly suspected. The dawn of the aseptic ideal in surgery and obstetrics has given a quite new significance to the conception of cleanliness. From the point of view of the aseptic ideal we are all unclean in person, dress, and environment, our foods are all more or less poisonous, and the state of our great centres of population is unspeakably filthy throughout. Imagine the horror with which we should regard a revival of the habits of those mediæval grandees who looked upon a bath as a thing to be taken once or twice in a lifetime at the most, and then with no slight misgivings as to the possibility of a fatal result. Even such is the emotion proper to the mind of the medical pioneer, who, aspiring to cleanliness in the twentieth century sense of the word, beholds our native land as it is. Obviously personal cleanliness in the scientific sense is impracticable without social cleanliness, or in other words a truly rational civic hygiene as its basis. Dust must be eliminated by an appropriate treatment of our streets and roads, houses must be spaced out, slums abolished, railways electrified, furniture and upholstery reconsidered, the dress of men and women revolutionised. Some three years ago we had an unusually wet summer, and, as you no doubt remember, we doctors had little or nothing to do. Never had there been such a dearth of seasonable ailments; and if part of this immunity was due to the free flushing of sewers, part may also be attributed to the absence of germ-distribution by dust. The time seems ripe for the formation of a league of medical pioneers vowed to a crusade against the dirt and noise which are the twin curses of our modern civilisation.

The subject of noise is indeed one upon which I could speak feelingly, seeing that I am at present living in the close vicinity of a shunting sideway, but the topic might prove conducive to unparliamentary language on my part, if not on yours. If my views as to the nature of modern obligations in the matter of cleanliness, as to its being practically synonymous with asepsis, appear Utopian, let me suggest that Science is nothing if not Utopian, for she aims at and will ultimately accept nothing short of perfection. Of course, I am aware that inasmuch as a certain percentage of dust is found in the air of mid-ocean, polar and mountain regions, its complete eradication from that of inhabited districts is in the strict sense impracticable. But such dust contains, I presume, no virulent micro-organisms, and can be inhaled without danger of incurring influenza, phthisis, rubeola or scarlet fever. In fact, dust in the scientific sense is one thing, and in the popular sense of mere dirt quite another.

As an example of what can be and has been done in an analogous line of activity by intelligent expert co-operation, let me refer you to a little book on *The Real Triumph of Japan*, by Dr. Louis Livingstone Seaman. In wars between civilised countries it has for centuries been the rule that, of the total mortality incurred, only 20 per cent. die on the battle-field or from the effect of wounds, the remaining 80 per cent. perishing from disease, mostly of a preventable kind. It is said that during the Crimean War the Allied Forces lost in six months 50,000 from disease and 2,000 from bullets. In the Boer War our own losses from disease were simply frightful, greater in proportion than those in the American Civil War, where nearly three times as many died of disease as of bullet or sword. Now Japan in her first great modern war, against China in 1894, followed pretty much the general rule, having 45 per cent. of her men incapacitated by disease. But Japan differed from other nations in that this object-lesson was not thrown away. She evolved a system of her own, based on the best available models, but considerably modified, and aiming at prevention rather than cure. The result was that in the great war just brought to a triumphant conclusion, against every four casualties of battle the Japanese had but one from disease, thus completely reversing the traditionary proportion. The expert had been given a free hand, and the expert had justified his existence.

If results at all comparable to this could be obtained in reduction of infant and adult mortality in civil life, it would pay the community to subsidise the entire profession on condition of its organisation as a society of hygienic experts for the prevention as well as the cure of disease. Our public health service is no doubt admirable so far as it goes; it may some day be supplemented by a private health service, a service of general practitioners—preventive rather than curative in aim. Why await the advent of mischief which could so often have been averted by the detection and removal of the causes which must bring it about? We shall never do justice to our knowledge until we are, at least in some degree, emancipated from direct economic dependence upon the voluntary requisition of our patients. We are often constrained to give medicine where we should prefer to withhold it, because we cannot afford to combat the superstition that recovery, apart from the ingestion of drugs, is necessarily slow and incomplete. The health of a nation, which is its vital capital, is after all made up of that of the private individuals who compose it, and though much may be done by efficient drainage, pure water supply, meat inspection, visitation of dairies, prevention of adulteration, notification and removal of infectious cases, and other public means, to prevent the grosser forms of epidemic and endemic disease, there is an immense field for fruitful effort in the detection and combating of those minor sins against personal hygiene which are the source of innumerable ailments. Is not the rapid increase of lunacy strikingly suggestive of ill-regulated households, erroneous dietetics, neglect or abuse of exercise, frenzied pursuit of pleasure and excitement, as contributory, and quite preventable, causes? Might not a word in season to someone in authority often have averted the tragic issue?

Then there is the stupendous evil of infant mortality, with which, considering the importance of the constitutional factor, known to the family doctor and to none beside, I am convinced no cut-and-dried public advice and regulations can effectively deal. Infant feeding is pre-eminently a relative matter; there is no possibility of laying down a definite percentage of nutrient materials that shall suit every artificially-fed infant. As things are at present, we are too often called in too late to remedy the results of malnutrition. I think that every mother should suckle her child unless exempted by a certificate of unfitness to do so. And I further suggest that on registering the birth of a child the parent should state whether it is to be naturally or artificially fed. If the latter, a form should be supplied to be filled in at definite intervals by the medical attendant. On this form periodical entries might be made by him, giving particulars of the dietary and progress of the infant. In this way valuable statistical evidence would be collected as to the best way of rearing hand-fed children. All such information should be paid for out of the public funds. In some such way the national disgrace of our present infant mortality might be effectually removed.

In conclusion, I submit that the doctor, both as expert and pioneer, has by no means the power and authority that are his due. Of influence we have plenty; and influence is in a way a higher and greater thing than authority. We need not forfeit that, but we must have the other also. We must come out into the open, make our voices heard above the futile clamour of partisans, clear the cranks and babblers out of the path of progress, point the way to higher and saner ideals. Point the way? Nay, more, we must lead, for example is better than precept. Such a function is worth the sacrifice of some of our official conservatism, some of our diffidence and reserve, even, at a pinch, of the outermost husk of our cherished respectability.

